# Analysis of Anterior Trunk Symmetry Index (ATSI) in healthy school children based on 2D digital photography: normal limits for age 7-10 years

**DOI:** 10.1186/1748-7161-8-S2-P10

**Published:** 2013-09-18

**Authors:** Lukasz Stolinski, Dariusz Czaprowski, Mateusz Kozinoga, Krzysztof Korbel, Piotr Janusz, Marcin Tyrakowski, Katsuki Kono, Nobumasa Suzuki, Tomasz Kotwicki

**Affiliations:** 1 Rehasport Clinic, Poznań, Poland

## Background

Digital photography for a 2-dimensional assessment of the body shape is a valuable method to both document the human posture and calculate the main quantitative parameters of it. 

## Purpose

The goal of this study was to assess the frontal plane symmetry of the anterior trunk in healthy school children based on the digital photography by measurement of the Anterior Trunk Symmetry Index (ATSI). .

## Methods

The study comprised 421 school children, both sexes, aged 7-10 years, with no clinical evidence of scoliosis (Angle of Trunk Rotation <5º). One frontal photograph of anterior trunk in spontaneous standing position was taken with a digital camera in standardized manner. The semi-automatic software for calculation of photogrammetric parameters was developed in collaboration with an IT specialist. The photographs were analyzed to obtain a quantitative assessment of the ATSI parameter. The intra-observer error was calculated by the first author by measuring the pictures of 14 children three times, selected randomly, at the interval of at least two days. The inter-observer error was calculated by one surgeon and two experienced physiotherapists by measuring the pictures of 60 children, selected randomly. The normal upper value limit was calculated as mean + 2SD. 

## Results

The mean ATSI value for 421 children was 24.3. ±12.7 (girls 24.4.±12.1, boys 24.0.±13.5). The ATSI values for each age group were: (1) 7-year-old children (N=117): 26.0 ±12.9 (girls 25.9 ±12.5, boys 26.2 ±13.4); (2) 8-year-old children (N=85) 23.3 ±13.7 (girls 23.0 ±12.2, boys 23.7 ±16.1); (3) 9-year-old children (N=109) 24.5 ±12.7 (girls 23.9 ±11.9, boys 26.1 ±14.1); and (4) 10-year-old children (N=110) 22.9 ±11.7 (girls 24.5 ±11.6, boys 21.8 ±11.5). For all age groups, the mean ATSI for boys did not differ significantly from the mean ATSI for girls (P>0.05). For both boys and girls, the mean ATSI did not differ among the four age groups (P>0.05). The intra-observer error was 1.07. The inter-observer error for the three observers was 4.06. The upper value limit was: (1) for 7-year-old children: girls=50.9 and boys=53.0; (2) for 8-year-old children: girls=47.4 and boys=55.9; (3) for 9-year-old children: girls=49.9 and boys=47.7; and (4) for 10-year-old children: girls=47.7 and boys=44.8. 

## Conclusions and discussion

Using semi-automatic software, the ATSI parameter could easily be calculated on regular digital photographs. The mean value of ATSI did not differ between boys and girls for the age group range of 7-10 years. Clinical usefulness of the ATSI parameter is yet to be determined by undertaking studies on larger groups of healthy and scoliotic children at different ages. 

## References

[B1] Stolinski L Kotwicki T Czaprowski D Chowanska J Suzuki N Analysis of the Anterior Trunk Symmetry Index (ATSI). Preliminary report Stud Health Technol Inform 2012 176 242 246 22744500

